# Extracellular Vesicles in Asthma: Intercellular Cross-Talk in TH2 Inflammation

**DOI:** 10.3390/cells14070542

**Published:** 2025-04-03

**Authors:** Naila Arif Cheema, Annalisa Castagna, Francesca Ambrosani, Giuseppe Argentino, Simonetta Friso, Marco Zurlo, Ruggero Beri, Matteo Maule, Rachele Vaia, Gianenrico Senna, Marco Caminati

**Affiliations:** 1 Department of Medicine, University of Verona, Piazzale L.A. Scuro, 37134 Verona, Italy; nailaarif.cheema@univr.it (N.A.C.); marco.zurlo1@gmail.com (M.Z.); rachele.vaia@univr.it (R.V.); gianenrico.senna@univr.it (G.S.) annalisa.castagna@univr.it (A.C.); francesca.ambrosani@univr.it (F.A.); giuseppe.argentino@univr.it (G.A.); simonetta.friso@univr.it (S.F.); ruggero.beri@univr.it (R.B.); 2 Allergy Unit and Asthma Center, Verona Integrated University Hospital, 37126 Verona, Italy

**Keywords:** asthma, Th2, T2 inflammation, extracellular vesicles, exosomes, personalized medicine, intercellular crosstalk, immune cells, epithelial cells

## Abstract

Asthma is a complex, multifactorial inflammatory disorder of the airways, characterized by recurrent symptoms and variable airflow obstruction. So far, two main asthma endotypes have been identified, type 2 (T2)-high or T2-low, based on the underlying immunological mechanisms. Recently, extracellular vesicles (EVs), particularly exosomes, have gained increasing attention due to their pivotal role in intercellular communication and distal signaling modulation. In the context of asthma pathobiology, an increasing amount of experimental evidence suggests that EVs secreted by eosinophils, mast cells, dendritic cells, T cells, neutrophils, macrophages, and epithelial cells contribute to disease modulation. This review explores the role of EVs in profiling the molecular signatures of T2-high and T2-low asthma, offering novel perspectives on disease mechanisms and potential therapeutic targets.

## 1. Introduction

**Figure 1:**
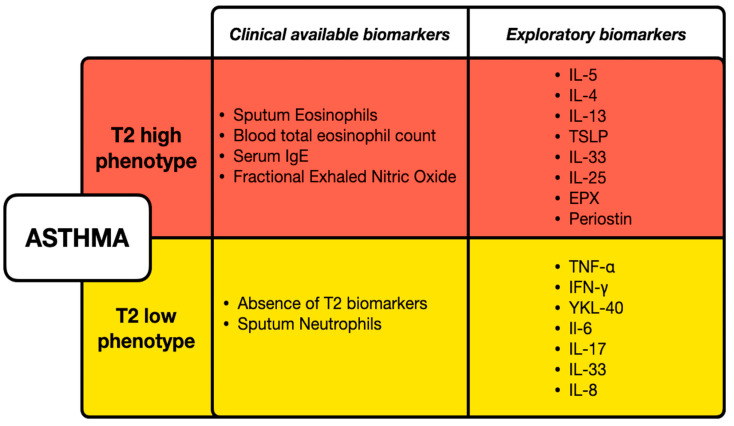
Clinical and exploratory biomarkers for T2-high and T2-low asthma phenotypes. T2-high asthma is characterized by elevated eosinophils, serum IgE, and fractional exhaled nitric oxide, with additional exploratory biomarkers including serum/plasma concentration of IL-4, IL-5, IL-13, and Periostin. T2-low asthma is defined by the absence of T2 biomarkers and the presence of sputum neutrophils, with exploratory markers such as serum/plasma concentration of TNF-α, IFN-γ, and IL-6, IL-17, IL-33, IL-8.

Asthma is a heterogeneous chronic airway condition, characterized by bronchial hyperreactivity, airway obstruction, and inflammation [1]. Based on the underlying cellular and molecular mechanisms, different asthma endotypes have been described, encompassed within a broader classification including T2-high asthma (allergic/eosinophilic) and T2-low asthma (non-eosinophilic) [2]. The T2-high endotype includes early-onset allergic asthma, late-onset eosinophilic asthma, and Aspirin-exacerbated respiratory disease (AERD) [3], while the non-T2 endotype comprises phenotypes like obesity-related asthma and late-onset neutrophilic asthma [4]. The regulation of asthma phenotypes involves both innate and adaptive immune responses, with immune cells playing a critical role in the pathogenesis of asthma [5]. Epithelial cells and dendritic cells (DCs) are particularly significant, influencing the activation and differentiation of T helper cells through cytokine secretion and antigen presentation [6,7]. This complex interplay between environmental factors, immune mechanisms, and cellular actions underlines the importance of identifying asthma based on endotypes [8]. However, the number and accuracy of biomarkers available in daily clinical practice are limited. Serum immunoglobulin E (IgE), blood eosinophils, and exhaled nitric oxide (FeNO) contribute to identify T2-high asthma, whilst their absence defines a T2-low endotype. Figure 1 displays the available clinical biomarkers for T2-high and T2-low phenotypes, which have been identified based on recent literature [9,10]. Therefore, discovering new clinical and measurable biomarkers for asthma endotypes can provide deeper understanding into the mechanisms of the disease and eventually support personalized treatment approaches for patients [11].

In this scenario, extracellular vesicles (EVs) are gaining growing importance as they play pivotal roles in cell-to-cell communication and distal signaling activation. EVs are membranous vesicles secreted by almost every kind of cell. They can be subdivided into different classes according to their function, size, and origin. Exosomes are a family of EVs characterized by small size (mean diameter ranging from 40 to 160 nm ca) and originating from late endosomes. Exosomes are the most studied type of vesicles, either for elucidating pathophysiological mechanisms or for disease treatments. The concept of “exosome” was first introduced by Rose Johnstone as a way to better understand the biological transformation of a reticulocyte into a mature red blood cell [12]. From this point on, EVs are commonly referred to by the authors of this review as exosomes. EVs are present in various body fluids, including plasma, urine, semen, bronchial fluid, synovial fluid, tears, and milk, highlighting their widespread role in intercellular communication [13]. EVs have been extensively studied in various physiological and disease status, including chronic respiratory disease [14,15], highlighting their potential as diagnostic/prognostic biomarkers. EVs originate from the inward folding of the cell membrane through endocytosis, followed by the formation of multivesicular bodies (MVB), secreted via exocytosis, and they can modulate various immune-regulatory pathways [16,17]. They are characterized by a lipid bilayer membrane surface composed by high concentrations of lipid biomolecules, including phosphatidylserine (PS), sphingomyelin, cholesterol, and ceramides [18,19,20]. Within the lipid bilayer membrane, EVs and in particular exosomes carry tetraspanins (CD9, CD63, CD81), adhesion molecules, and major histocompatibility complex (MHC) antigens from both class I and class II [21]. As exosomes originate from endosomes, they are are marked by cellular origin-specific proteins like ALIX, tumor susceptibility gene (*TSG101*), flotillin involved in MVB formation, and proteins (annexins, Rabs, and GTPases) essential for membrane transport and fusion [22]. EVs and exosomes also carry various proteins inside, along with potential metabolic enzymes, nucleic acids, consisting of DNAs, mRNAs, microRNAs (miRNAs), long noncoding (lnc) RNAs, and circular (circ) RNAs [23,24]. Certain proteins found in exosomes are unique to their originating cells, whereas others remain constant regardless of the cellular origin [25]. RNA sequencing has shown that miRNAs dominate RNA content, especially in human plasma-derived exosomes, representing over 42.32% of total sequencing reads and 76.20% of reads aligning to recognized sequences [26,27]. The peculiar characteristics of EVs, such as stability, accessibility, biosafety, molecular cargo, surface antigens, etc., make them useful indicators of disease severity and progression, offering opportunities for their use in investigating the differences between asthma phenotypes but also in developing targeted and effective therapeutic approaches [28]. EVs have been extensively investigated to explore their links to the development of inflammatory and various respiratory conditions [29], leading to important comprehension of the cellular cross-talk mechanism of allergic diseases [30,31]. They are also implicated in the disrupted signaling processes associated with allergies and allergic asthma, underscoring their significant contribution to disease processes [32]. EVs possess diverse functions within the immune system, encompassing the expression of several genes, regulation of immune responses, presentation of antigens, promotion of antitumor immunity, and inhibition of immune system activity [33]. Moreover, they are crucially involved in the development of specific immune-related disorders, including asthma, underlining their impact on immune system dynamics [34]. Moreover, they contribute to tissue remodeling by delivering growth factors and miRNAs that promote healing and regeneration [35]. Their functions in both sustaining homeostasis and promoting immunomodulation make them a focal point of research for novel insight on the knowledge of asthma endotypes [36]. In this review, we will explore the potential relevance of EVs, particularly derived from cells of the immune system, or related to it, involved in asthma pathophysiology, in profiling different asthma endotypes and phenotypes, particularly focusing on T2-high vs. T2-low molecular fingerprints.

## 2. The Cellular Drivers in Asthma Inflammation and EVs’ Related Role

Bronchial inflammation in asthma reflects the impaired activation of the immune system when exposed to environmental determinants or endogenous triggers. Although some pathobiological aspects are still controversial, recent research advances have highlighted that in genetically predisposed individuals, increased epithelial susceptibility of the airways accounts for the initiation of the immune response, including a number of different cells and cytokines, as detailed below. In allergic subjects, it is the case of the well-known type 1 IgE-mediated hypersensitivity, but similar Th2 inflammation may also be triggered in non-atopic individuals by different environmental stimuli including pathogens and pollutants [37]. The relevance of innate immunity in polarizing a Th2 impaired response in asthmatic patients has also been highlighted. Under that perspective, the complex cross-talk between airway epithelia, innate and adaptive immunity is emerging as a major driver of Th2 inflammation [37,38]. Dysfunctional epithelial cytokine activation is nowadays considered as the background of the Th2-low pattern, although recruiting cells and cytokines other than the Th2 context [39].

**Figure 2:**
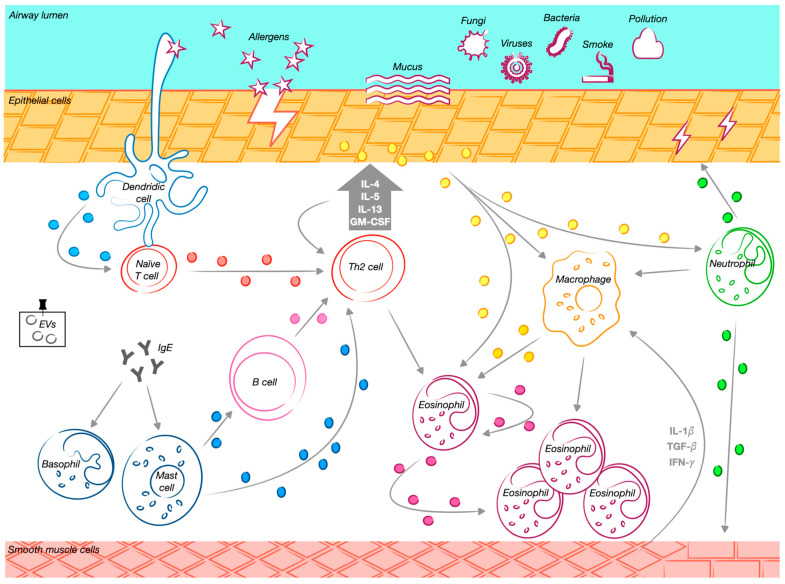
The role of extracellular vesicles and their cellular progenitors in asthma. Exposure to various external stimuli triggers a Th2-driven immune response in the airways. Mature dendritic cells promote the differentiation of naïve CD4⁺ T cells into Th2 cells, initiating a cascade of immune modulation. Extracellular vesicles (EVs) released by different immune cells contribute significantly to this process. Dendritic cell-derived EVs facilitate T cell activation and function as antigen-presenting structures. Macrophage-derived EVs carry active enzymes involved in inflammation, supporting leukotriene synthesis and attracting granulocytes to inflamed areas. Mast cell-derived EVs promote B cell activation, stimulating their proliferation and influencing other lymphocytes. Eosinophil-derived EVs enhance pro-inflammatory activity by increasing reactive oxygen species production and recruiting additional eosinophils to sites of inflammation. T cell-derived EVs contribute to immune activation by promoting Th2 cytokine secretion, while B cell-derived EVs present major histocompatibility complex class II and costimulatory molecules, essential for T cell responses. Additionally, airway epithelial cell-derived EVs transport bioactive molecules that regulate inflammation, stimulate monocyte proliferation, and increase cytokine and leukotriene production. This process exacerbates airway hyperresponsiveness and promotes the accumulation and activation of macrophages, neutrophils, and eosinophils in the airways. The release of these EVs is further amplified by Th2 cytokines, particularly IL-13, reinforcing the inflammatory cascade characteristic of allergic airway diseases. Neutrophil-derived EVs also play a crucial role in airway remodeling by modulating structural changes in smooth muscle cells. Through matrix metalloproteinase-9 (MMP-9) activity, these EVs disrupt epithelial cell junctions and contribute to airway smooth muscle remodeling, further exacerbating disease progression.

EVs play a role in asthma inflammation development, as they are released by critical asthma-related cells like eosinophils, mast cells (MCs), DCs, T cells, neutrophils, macrophages, epithelial cells, etc. EVs can be considered dynamic biomarkers within the context of cell-to-cell cross-talk, as the molecular structures expressed on their surface can inform about the cells originating the EVs and what the EVs are targeting. In addition, the reach repertoire EVs contain can be related to the “message” they are carrying out within the context of the ongoing inflammation. In the following sections, we will highlight the contributions of different cell types in terms of EVs and their role in asthma, as schematically illustrated in Figure 2 and Table 1. 

### 2.1. TH2-High Asthma-Related Cells

#### 2.1.1. Epithelial Cell-Derived EVs

The strategic position of epithelial cells at mucosal sites demands continuous interaction with basolateral immune cells for tissue homeostasis. Thus, exosomes secreted from mucosal epithelial cells mediate this critical exchange of cellular signals. The breakthrough understanding that the airway epithelium plays a crucial role in triggering and directing T2 adaptive immune responses against allergens marks a significant development in the study of allergy and asthma [40]. It has been found that the airway epithelium secretes cytokines which act as “alarms” to the immune system, leading to the activation of DCs, which guides the naive T cells to differentiate into Th2 cells. This process is influenced by “alarmins” such as TSLP [41], granulocyte–macrophage colony-stimulating factor (GM-CSF), IL-33, IL-25, and supplementary factors like serum amyloid A protein [42]. Further immunohistochemical studies on lung tissue have shown that exosomal markers (CD63, CD81, and Rab-5b) are predominantly found in bronchial epithelial cells (BECs) and also in macrophages. This indicates that BECs play a pivotal role in enhancing exosome-driven cell–cell communication inside the lungs. Kulshreshtha et al. also found that exosomes associated proteins are elevated in the lungs of asthmatic mice as compared to wild-type mice. In the context of asthma-related inflammation, there is an elevated secretion of exosomes specifically from epithelial cells, stimulated by IL-13. Moreover, the exosomes originating from BECs promote the proliferation and infiltration of undifferentiated macrophages [43]. Kesimer and Gupta showed that airway epithelial cell (AEC)-derived exosomes are found to have extensive, intertwined filamentous formations on their surfaces, specifically membrane-bound mucins, leading to airway inflammation remodeling. Through various methods, this study demonstrated that in culture, airway epithelial cell-derived exosomes carry mucins of different sizes attached to their membranes, which can modify the physical attributes of these structures. This alteration impacts their measured dimensions, shape, and electrical charges [44]. The effects of exosomes derived from bronchoalveolar lavage fluid (BALF) were evaluated in another study which showed that these exosomes can activate airway epithelial cells, causing inflammation by increasing the secretion of IL-8 and LTC4 [31]. In a recent study, it was demonstrated that exosomes secreted from OVA-induced AECs containing lnc-TRPM2-AS promote asthma development by contributing to the differentiation of CD4+ T cells into Th2-like cells. Ephenedrine, a sympathomimetic drug, which function as both an α- and β-adrenergic agonist, has been wildly used for asthma treatment and it is able to reverse this effect, making it quite interesting to target AEC-derived exosomal lnc-TRPM2-AS in treating asthma [45]. Zhang et al. showed that AEC-derived exosomes are key in controlling allergic responses mediated by DCs. Specifically, exosomes with CNTN1 protein enhance the recruitment, proliferation, differentiation, and activation of monocyte-derived DCs, relying on the Notch2 receptor. Furthermore, targeting the CNTN1-Notch2 pathway offers a way to influence both the innate and adaptive immune responses. Therefore, the significant rise in CNTN1 levels in asthma patients confirms its critical role, aligning with previous observations in cell cultures and mouse models [46]. A new direction for diagnosing and treating airway conditions was proposed, focusing on identifying the origins and mechanisms behind the presence of tissue factor (TF) in the lung microenvironment. Researchers found that mechanical stress on airways induces the expression of TF mRNA and the production of TF proteins and exosomes containing TF in normal BECs [47]. This suggests that exosomes can transport TF between different cell types through vesicle trafficking, linking it to asthma, which involves angiogenesis beneath the epithelium [48,49]. Recently, Park et al. demonstrated that asthmatic patients present higher levels of TF RNA expression in the basal layer of the airway epithelium. TGF-β was shown to play an important role in mechanical compression and hence in inducing tissue factor expression in BCE cells, showing its involvement in cellular response to mechanical stress in asthmatic patients [50]. Researchers found that while fibroblast TGF-β2 levels were consistent between healthy individuals and asthmatics, its expression within exosomes was reduced in those secreted from severe asthmatic patients. These exosomes boosted epithelial cell proliferation in both groups more than the control [51,52]. Increasing TGF-β2 in fibroblast-derived exosomes of severe asthma patients reduced the proliferation of epithelial cells, and reducing it had the opposite effect, suggesting a regulatory role of TGF-β2 in exosome-mediated cell proliferation [52]. In a recent study, primary normal human bronchial epithelial (NHBE) cells were found to release EVs both apically and basally. In particular, IL-13 treated cells secrete EVs with decreased levels of miRNA, in particular miR-34a, miR-92b, and miR-210, which seem to play a pivotal role in triggering an early Th2 immune response, contributing to the initiation and development of asthma [53]. MiR-34a has been described to regulate the functions of dendritic cells and their maturation targeting the Wnt pathway. Its levels increase in normal DCs and decrease after antigen presentation, and its overexpression is found to be associated with the suppression of CD4+ T cell polarization [54]. MiR-92b is involved in epithelial-to-mesenchymal transition (ETM), which is a feature of asthma [55]. MiR-210 is involved in the regulation of T cell function targeting FOXP3 [56].

#### 2.1.2. Eosinophilic-Derived EVs

Eosinophils, critical to inflammatory and immune responses of asthma, are influenced by T2 immune cytokines (like IL-4, IL-5, IL -13), chemokines (like eotaxins) [57], cysteinyl leukotrienes (cysLTs) (LTC4, LTD4, and LTE4), and granule mediators. Eosinophil cytotoxic proteins include major basic protein (MBP), eosinophil cationic protein (ECP), eosinophil peroxidase (EPX), and eosinophil-derived neurotoxin (EDN), driving their development, survival, and migration [58]. These cytokines, released by Th2 lymphocytes and ILC2s, recruit eosinophils to the lungs, where they contribute to asthma hallmarks such as airway remodeling, mucus hypersecretion, and inflammation through the action of granule proteins and the secretion of several cellular mediators including metalloproteinases and cytokines [59]. The complexity of eosinophil function and their contribution to asthma pathophysiology highlight the crucial role of eosinophil-derived exosomes in mediating immune responses and structural changes within the lungs, highlighting their potential as therapeutic target in asthma [60,61]. Eosinophil-derived exosomes play a significant role in asthma pathogenesis, with studies showing that both healthy individuals’ and asthma patients’ exosomes have distinct impacts on disease progression [36]. Specifically, EVs from asthmatic patients’ eosinophils increase the production of nitric oxide (NO) and reactive oxygen species (ROS), enhance chemotaxis, and upregulate cell adhesion molecules like ICAM-1 [62] and integrin α2 on eosinophils’ surfaces [63]. These mechanisms contribute to the inflammatory response and structural changes in the lungs, including increased epithelial cell apoptosis, impaired wound healing, as well as stimulated muscle cell proliferation linked with ERK1/2 phosphorylation [64,65]. Additionally, the presence of these exosomes alters gene expression in various cell types, indicating a significant role in asthma pathology by influencing structural lung cells [63,64]. The journey to uncovering the ability of exosome secretion of eosinophils started in early 2002 with the identification of the CD63 surface marker within eosinophils, linked to piecemeal degranulation upon interferon-gamma (IFN-γ) stimulation [66]. Moreover, it was discovered that eosinophils express CD9, another exosomal marker, which, along with MHC class II, resides in detergent-resistant membrane microdomains (DRMs) following activation by granulocyte–macrophage colony-stimulating factor (GM-CSF), providing eosinophils with the capability to act as antigen-presenting cells (APCs) [67].

Mazzeo et al., by studying exosomes secreted from eosinophils isolated from the peripheral blood of both asthmatic patients and healthy controls, confirmed previous findings regarding EV secretion by asthmatic patients and also showed that stimulation with IFN-γ significantly enhanced exosome production. Additionally, they demonstrated that patients with asthma exhibit increased production of exosomes, suggesting a potential link between exosome secretion and asthma pathogenesis [61]. Other researchers confirmed that eosinophils from asthmatic individuals produce a greater quantity of exosomes compared to healthy subject-derived eosinophils and also showed that those eosinophils have a decreased apoptosis rate [63]. Exosomes notably increase the production of NO and ROS along with enhancing the chemotaxis and adhesion of eosinophil, suggesting their role in promoting inflammation-related mechanisms associated with asthma [68]. Another study showed that when bronchial smooth muscle cells (BSMCs) were co-cultured with eosinophil-derived exosomes, there was a marked increase in BSMC proliferation and the upregulation of genes such as *VEGF-A* and *CCR3*, important for angiogenesis and fibrotic activity. This evidence emphasizes the significant contribution of eosinophil-derived exosomes to the complex pathophysiological state of asthma, highlighting their crucial role in both the inflammatory response and structural changes within the airways, such as hyperplasia and hypertrophy of BSMCs [64].

#### 2.1.3. Lymphocyte-Derived EVs

Lymphocytes play a crucial role in inflammation related to allergies and asthma. B lymphocyte cells are essential in initiating allergic sensitization and the adaptive immune response associated with asthma [69,70]. Following the Th2 activation and the subsequent Th2 cytokines secretion, B lymphocytes generate antigen-specific immunoglobulin E [71]. Exosomes originating from B cells exhibit the features of their originating cells, including the presence of MHC classes I and II, integrins β1 and β2, and the costimulatory molecules CD40, CD80, and CD86 [72]. Consequently, they can present antigen peptides to T cells, thereby inducing T cell responses and the release of the proinflammatory cytokines IL-5 and IL-13 [73]. Additionally, B cell exosomes present HSP70, which plays an important role in DC maturation [74]. Admyre et al. further demonstrated that B cell-derived exosomes are capable of presenting allergen-derived peptides, thereby inducing allergen-specific T cell proliferation and stimulating cytokine secretion. These authors also suggest that in allergic immune reactions, the APC-derived exosomes are significant contributors to T cell activation [75]. The precise mechanisms through which B cell-derived exosomes activate T cells remain a matter of debate. There are some studies suggesting that exosomes might directly stimulate T cells [76], while other studies proposed that the efficacy of exosomes in modulating T cell responses requires the involvement of APCs [77,78]. This dichotomy emphasizes the complex nature of exosome-mediated cell-to-cell interaction in the immunological response, particularly in asthma and other allergic reactions [79]. B lymphocyte-produced exosomes not only carry APC molecules that are responsible for antigen presentation to T cells but also stimulate the release of cytokines IL-5 and IL-13 [80,81]. T lymphocytes, including activated CD4+ T cells, can secrete exosomes upon activation [29,82]. T cell-derived exosomes trigger MC activation and degranulation, resulting in cytokine release, tissue remodeling, and increased airway reactivity [83]. These exosomes contain a range of cellular protein markers such as CD25, T cell receptor (TCR), and CD4, along with liposomal-associated membrane protein 1 (LAMP1) and lymphocyte function-associated antigen-1 (LFA1), playing a role in modulating the immune response by inhibiting CD8+ T lymphocyte activity [84]. Inflammation derived from Th2 cells, which leads to increased airway sensitivity and structural changes in the airways, represents one aspect of asthma [59,85]. Shefler et al. found that T cell EVs carry signaling molecules similar to their cells of origin and are able to activate MC degranulation and the release of cytokines, suggesting a role of T cell EVs in promoting MC activation at distant inflammation sites [86]. A recent study illustrated a possible role of EVs in cytokine-based communication during lung inflammation. Results showed that EVs derived from Th2 cells prolong eosinophilia and promote eosinophil survival through the action of IL3 cytokine present on their surface, which inhibits apoptosis via the activation of Jak1/2-dependent pro-survival programs [87].

#### 2.1.4. Mast Cell-Derived EVs

MCs, characterized by large granules, are found throughout vascularized tissues close to blood vessels [88], smooth muscle cells, mucous glands, and hair follicles [89]. These cells, which derive from hematopoietic stem cells, contain numerous granules rich in histamine and heparin [90,91]. MC-derived EVs and exosomes have a diverse composition, encompassing a variety of elements such as CD13, Cdc25, ribosomal protein 6 kinase, phospholipases, HSPs, annexin V, immune-related factors like MHC class II, co-stimulatory molecules (CD86, CD40, and CD40L), and adhesion molecules such as LFA-1 and intercellular adhesion molecule-1, in addition to mRNAs, small RNAs, and miRNAs [92,93]. In asthma and comorbidities, EVs are significant in the pathogenesis or mitigation of disease symptoms and influence the inflammatory milieu. A recent study showed that mast cell-derived exosomes secrete miR-21, which is able to promote oxidative stress and inflammation in asthmatic mice via the DDAH1/Wnt/β-catenin axis, highlighting the potential therapeutic importance of targeting miR-21 inhibitor and mast cell-derived exosomes in asthma treatment [94]. Skokos et al. illustrated that exosomes from bone marrow-derived mast cells and MC lines (P815 and MC9) contain immunologically significant molecules such as MHC class II, LFA-1, ICAM-1, and CD86. This suggests that MC-derived exosomes could contribute to the mobilization of B and T cells into the lungs [95]. Further research conducted by Valadi et al. showed that exosomes from human MCs contain a unique set of mRNA and miRNA, revealing that MCs can exchange RNA with each other through EVs [96]. Specifically, MC-derived exosomes enhance the ability of DCs to present antigens to T cells and regulate T lymphocyte activation, leading to the secretion of IL-6, IL-12, and IFN-γ cytokines [29]. Moreover, it was found that exosomes from bone marrow-derived mast cells have an anti-IgE effect, lowering IgE levels and blocking mast cell activation. In murine models of allergic asthma, exosomes showed reduced airway inflammation, improved airway hyperresponsiveness (AHR), and affected remodeling in chronic asthma [97]. Additionally, it has been demonstrated that exosomes secreted by MCs play a role in regulating oxidative stress. According to a study by Eldh and colleagues, exosomes from MCs subjected to oxidative stress can convey a protective message to other cells facing similar stress, thereby decreasing cell mortality. The mRNA composition of these exosomes was significantly different from that within the originating cell and from exosomes generated by cells grown in standard conditions [98]. Moreover, a specific miRNA, miR-21, released by exosomal MCs, was shown to enhance oxidative stress and trigger inflammatory reactions in asthmatic mice through the DDAH1/Wnt/β-catenin signaling pathway [94]. In a recent study by Yang et al., the authors discovered that MC-derived exosomes activated by IgE exacerbate atherosclerosis by inducing endothelial dysfunction via the circular RNA CDR1as in asthma-mediated atherosclerosis patients. This finding could offer new understanding into how asthma influences atherosclerosis and suggests potential targets for diagnosis and treatment [99].

#### 2.1.5. Dendritic Cell-Derived EVs

Dendritic cells are key effector cells and the vital APCs in the immune system, responsible for capturing antigens, processing them, and presenting them to T lymphocytes, as well as playing a crucial role in initiating innate immunity [100]. TSLP, a cytokine produced by AECs following allergen exposure, stimulates the maturation of immature DCs into their mature form, which then travel to the lymphatic drainage nodes [41]. The engagement between DCs and T lymphocytes begins with the interaction of MHC class I/II molecules on DCs with the TCR on T lymphocytes [101] and is enhanced by secondary signaling interactions like OX40L-OX40, ICAM-1-LFA-1, and CD80/86-CD28 [102]. Depending on the specific antigens and cytokines involved, DCs can direct the differentiation of T lymphocytes into various subsets, including Th1, Th2, Th17, or regulatory T cells (Tregs), effectively influencing T lymphocyte polarization, which indicates that TSLP can induce DCs to activate the polarization of naïve CD4+ T cells to Th2 cells, developing a Th2 asthma phenotype [103] via OX40L [41]. DC-derived exosomes can activate allergen-specific Th2 cells through the presence of costimulatory molecules on their surface and show important roles in asthma modulation l [29]. Wahlund et al. investigated how during the intravenous administration of OVA-induced DC exosomes and microvesicles to mice, both types of vesicles displayed significant levels of immunostimulatory molecules. Despite this, only exosomes were able to specifically activate OVA-targeted CD8+ T cells and promote a higher production of OVA-specific IgG antibodies, outperforming MVs. This highlighted the higher concentration of OVA in exosomes than in MVs, explaining the superior immunogenic response triggered by exosomes [104]. Moreover, DC-derived exosomes were found to carry enzymes necessary for producing leukotrienes, the pro-inflammatory molecules involved in asthma pathogenesis, and deliver them to smooth muscle cells [105,106]. In addition, these exosomes were packed with chemotactic eicosanoids, which were observed to facilitate the migration of granulocytes in vitro and promote inflammation [106]. Recent findings have identified various subsets of pulmonary DCs, such as conventional type 1 cDCs (cDC1s), cDC2s, and plasmacytoids (PDCs), each contributing differently to asthma pathogenesis [107,108]. This discovery emphasized the importance of determining the source and impact of DC-derived exosomes according to their specific subtypes.

### 2.2. TH2-Low Asthma-Related Cells

#### 2.2.1. Neutrophil-Derived EVs

Neutrophils, associated with low Th2 response and severe asthma phenotypes, can secrete exosomes that contribute significantly to asthma onset [109]. Recent research proposed that neutrophils play a crucial role in inflammation [110] and the proliferation of asthma despite the small number of studies reported in the literature [111]. Neutrophils are involved in altering the pathophysiology of airways, e.g., chemokines produced by neutrophils recruit monocytes and macrophages to the airways, modifying the inflammatory response [112]. High neutrophils in T2-low asthma led to increased airway smooth muscle (ASM) sensitivity, and exosomes from neutrophils play a role in regulating changes in ASM structure and remodeling [113,114]. In a study, it was shown that OVA-induced airway epithelium-derived exosomes significantly increase airway hyper-responsiveness and trigger the accumulation or activation of macrophages, neutrophils, and eosinophils in the airways [115]. Furthermore, it was demonstrated by Butin-Israeli et al. that neutrophil EVs have MMP-9 activity, leading to the breakdown of tight junction proteins and consequently the disruption of epithelial cell connections [116]. Recent research has indicated that exosomes released by neutrophils in response to lipopolysaccharide (LPS) can promote the migration and proliferation of ASMCs, playing a role in the structural changes seen in the airways of individuals with asthma [117]. Moreover, neutrophil-derived exosomes containing elastase significantly contributed to airway inflammation by proteolytic breaking down of the extracellular matrix, causing epithelial damage and helping in airway remodeling. Notably, the impact of encapsulated elastase was more potent than that of free elastase, underlining the critical role of exosomes in enhancing inflammatory responses [118].

#### 2.2.2. Macrophage-Derived EVs

Macrophages, key cells of the innate immune system, influence numerous functions such as tissue repair, immunomodulation, and angiogenesis [119,120]. They are classified into conventionally activated (M1) and activated (M2) groups, distinguished by specific cytokine profiles and cell surface markers [121]. M1 macrophages serve as initial defenders against intracellular pathogens and encourage the polarization of Th1 lymphocytes [122]. Representing about 70% of lung immune cells, macrophages [123] and monocytes are pivotal in managing airway inflammation and remodeling [124,125]. Research indicates that exosomes originating from macrophages might have a more significant impact on T1 immune reactions compared to T2 responses [126,127]. In severe steroid-resistant asthma (SSRA) exacerbations, M1 macrophages release high concentrations of inflammatory molecules (such as TNF-α, IL-1β, IL-6, iNOS), leading to neutrophil-dominated infiltration, increased AHR, and remodeling of the airways [128]. Moreover, Esser et al. also showed that exosomes from human macrophages have a proinflammatory role in asthma and that these exosomes contain enzymes for leukotriene biosynthesis, with a shift towards LTC4 production in exosomes compared to parent cells. TGF-β1 increases LTB4 in both cells and their exosomes, modifies cell and exosome surface markers, and reduces macrophage exosome yield [106]. Exosomes secreted by M2 macrophage reduce lung inflammation and asthma progression since they transport miR-370, which downregulates the expression of fibroblast growth factor 1 (FGM1) and inhibits the MAPK/STAT1 signaling pathway [129].

## 3. The Paradigm of Mesenchymal Stem Cell-Derived EVs

Mesenchymal stem cells (MSCs) have recently emerged as important for cell-based therapies and regenerative medicine and are widely studied in respiratory diseases such asthma [130]. Various sources of MSCs are known to display distinct properties, with bone marrow-, adipose tissue-, umbilical cord-, and placenta-derived stem cells being the most commonly used in current research. MSCs primarily exert their beneficial effects through EVs, with exosomes being the most well-characterized among MSC-EVs. Moreover, MSC-EVs exhibit therapeutic effects similar to MSCs but with reduced risks of immune rejection, tumorigenicity, and pulmonary embolism [131].

Mesenchymal stem cell-derived exosomes showed to have similar therapeutic effect to their parental cells [132] and have a potential role to replace them in asthma treatment by impacting the immune cells and inhibiting airway remodeling [13]. Research has demonstrated that adipose-derived stem cells (ASCs) and other MSCs can reduce allergic airway inflammation in bronchial asthma mouse models [133]. Additionally, ASC-conditioned media and ASC-derived extracellular vesicles have shown similar effectiveness to ASCs in alleviating allergic airway diseases. The immunomodulatory effects of ASC-derived EVs in allergic airway inflammation are thought to involve the suppression of Th2 cytokine production. Recent studies have also indicated that ASC-derived EVs can improve allergic airway inflammation in mouse models of asthma [134]. The systemic administration of human adipose tissue-derived mesenchymal stromal cells (AD-MSCs) showed beneficial effects on ovalbumin-induced allergic asthma, with a decrease in BALF total cells and eosinophils count, a decrease in IL-5 and TGF-β levels in lung tissue, and a decrease in CD3+CD4+ T cells in the thymus. The same effect was showed for AD-MSC-derived exosomes [135]. Numerous experimental and clinical studies have shown that the secretome produced by MSCs, which includes both soluble factors and EV-encapsulated components, plays a key role in their immunomodulatory and anti-inflammatory effects. As a result, the direct use of MSC-derived extracellular vesicles (MSC-EVs) has been proposed as a promising alternative to MSCs for treatments such as cartilage protection and asthma [136]. As a general statement, we can consider that MSC-EVs influence the biological activity of target cells by either directly activating surface receptors or delivering signaling molecules into cells. Many studies were conducted recently on the effect of MSC-EVs on inflammatory mechanisms and in particular asthma, both in vivo and in vitro. As an example, Xiang Li et al. showed that bone marrow-derived mesenchymal stem cell (BMMC)-derived exosomes highly expressed a specific miRNA, miR-223-3p, targeting the NLRP3 inflammasome known to be associated with high inflammation and the exacerbation of asthma. Specifically, they showed in OVA rats that miR-223-3p is able to regulate the NLRP3-induced ASC/Caspase-1/GSDMD signaling pathway, suggesting its possible role in protection against asthma [137]. MSC-derived exosomal miR-1470 is an interesting therapeutic target for asthma treatment since it induces the expression of P27KIP1 in asthmatic patients, promoting the differentiation of CD4+CD25+FOXP3+ Tregs [138]. A recent study showed that exosomes secreted from human bone marrow MSCs contain miR-188, which has a negative effect on airway remodeling and lung injury. It mitigates the pathological development of asthma with the modulation of the JARID2/Wnt/β-catenin pathway [139]. Furthermore, even other types of MSCs-EVs have been studied in the context of asthma research, such as umbilical cord mesenchymal stem cell-derived EVs (UCMSC-EVs). Exosomes from human umbilical cord mesenchymal stem cells (hUCMSCs) have a therapeutic effect in SSRA with an action on the NF-kB and PI3K/AKT signaling pathways. They promote macrophage M2 polarization and inhibit the expression of TRAF1 [140]. In a recent study, the effect of migrasomes, which recently identified EVs produced from migrating cells and involved in cell-to-cell communication, were evaluated in an asthma model. In particular, the inhibition of migrasomes secreted from hUCMSCs reduced the hUCMSCs’ anti-inflammatory action on OVA-induced animals, suggesting that migrasomes play a role in the protective effect of hUCMSCs in asthma. In fact, it was observed that migrasomes suppress the Th2 response induced by dendritic cells reducing airway inflammation and mucus secretion [141]. Xu et al. first demonstrated that nebulized hypoxic hUCMSC-EVs (Hypo-EVs) were able to reduce airway inflammation and remodeling in asthmatic mice [142] and then investigated their possible therapeutic effect on epithelial barriers. The authors suggested that the mechanism through which Hypo-EVs have a therapeutic effect on epithelial barriers both in vivo and in vitro could be the inhibition of p-STAT6 pathways after the delivery of CAV-1 to bronchial cells and increasing the expression of ZO-1 and E-cadherin [143]. In the context of asthma treatment, the therapeutic mechanisms of MSC-EVs can be categorized into a few key pathways: (i) MSC-EVs enhance the proliferation of regulatory T cells, strengthening immunosuppression, reducing eosinophil levels, and mitigating inflammation in asthma. (ii) They inhibit the activity of human type 2 innate lymphoid cells (ILC2s), thereby reducing Th2 cytokine levels, suppressing lung inflammation, and alleviating AHR. (iii) MSC-EVs facilitate the transition of macrophages from the pro-inflammatory M1 phenotype to the anti-inflammatory M2 phenotype. (iv) They contribute to the inhibition of airway remodeling. Chen and colleagues very recently proposed a nice review of the potential therapeutic use of MSC-EVs in the treatment of asthma. Moreover, compared to MSC treatment, MSC-derived EV-based cell-free therapy, based on ASC-, BMMC-, UCMSC-derived EVs, etc., offers several advantages, including enhanced safety, easier handling and storage, a lower risk of immune rejection, and no threat of vascular occlusion. However, several challenges, particularly related to the stability, efficiency, production, and quality control of EV content, as well as the delivery methods of MSC-EVs, continue to hinder the implementation of this approach for asthma treatment [144].

**Table 1:** Summary of research studies concerning cell-derived EVs and their contribution to asthma pathophysiology.

Cell Type	Main Findings	References
**Epithelial cells**	Exosomes derived from AECs can cause inflammation by increasing IL-8 and LTC4.	[31]
	BECs play a pivotal role in exosome-driven cell-to-cell communication and promote the proliferation and infiltration of undifferentiated macrophages.	[43]
	AEC-derived exosomes with intertwined filamentous formations on their surface lead to airway inflammation remodeling.	[44]
	Exosomes secreted by OVA-induced AECs promote CD4+ T cell differentiation in Th2-like cells.	[45]
	AEC-derived exosomes with CNTN1 protein play a significant role in modulating allergic responses via DCs.	[46]
	Mechanical stress leads to TF expression and its transport via exosomes in normal BECs.	[47]
	Exosomes can transport TF between different cells, linking it to asthma.	[48,49]
	TF expression levels were higher in asthmatic patients, and TGF-β plays an important role in asthma mechanical stress.	[50]
	TGF-β2 expression in exosomes was reduced in severe asthmatic patients.	[51,52]
	TGF-β2 secreted from exosomes has a regulatory effect in cell proliferation.	[52]
	Epithelium-derived exosomes secrete miRNA involved in asthma development.	[53]
	MiR-34a regulates the functions of dendritic cells and their maturation, targeting the Wnt pathway.	[54]
	MiR-92b is involved in epithelial-to-mesenchymal transition.	[55]
**Eosinophils**	Eosinophil-derived exosomes mediate immune responses and structural changes.	[60,61]
	Eosinophil-derived exosomes significantly influence asthma pathogenesis.	[36]
	EVs from the eosinophils of asthmatic patients increase NO and ROS production. Patients enhance chemotaxis, upregulate cell adhesion molecules, and upregulate integrin α2 in eosinophils.	[62,63]
	EVs from eosinophils contribute to the inflammatory response and structural changes in the lungs.	[64,65]
	Eosinophil-derived exosomes alter gene expression in various cell types, including lung cells, contributing to asthma pathology.	[63,64]
	Eosinophils express exosomal markers such as CD63 and CD9.	[66]
	The stimulation of eosinophils with IFN-γ enhanced exosome production, particularly in asthma patients.	[61]
	Eosinophils from asthmatic patients have a greater production of exosomes.	[63]
	Eosinophil-derived exosomes promote inflammation related with asthma.	[68]
	Eosinophil-derived exosomes contribute to airway structural changes.	[64]
**Lymphocytes**	B cell-derived exosomes exhibit the features of their originating cells and present HSP70, important for DC maturation.	[72,74]
	B cell-derived exosomes can present antigen peptides to T cells, inducing the release of proinflammatory cytokines.	[73]
	Antigen-presenting cell (APC)-derived exosomes are significant contributors to T cell activation.	[75]
	Two mechanisms through which B cell-derived exosomes activate T cells are direct stimulation and through the involvement of APC.	[76,77,78]
	B lymphocyte-produced exosomes stimulate the release of cytokines IL-5 and IL-13.	[80,81]
	Activated T cells release exosomes upon activation.	[29,82]
	T cell-derived exosomes trigger mast cell activation and degranulation, cytokine release, tissue remodeling, and increasing airway reactivity.	[83]
	T cell-derived exosomes inhibit CD8+ T lymphocyte activity.	[84]
	T cell-derived exosomes shape an optimal environment for immune cell operations, mediating communications to enhance immune response.	[59,85]
	T cell EVs are able to activate MC degranulation and the release of cytokines.	[86]
	Th2 cells promote eosinophil survival through the inhibition of apoptosis.	[87]
**Mast cells**	MC-derived exosomes carry immune-related factors, which play an important role in immunity.	[92,93]
	Mast cell-derived exosomes secrete miR-21, which promotes oxidative stress and inflammation in asthmatic mice.	[94]
	BMMC-derived exosomes could activate immune cells without direct contact, suggesting the mobilization of B and T cells into lungs.	[95]
	MCs can exchange RNA with each other through EVs.	[96]
	MC-derived exosomes enhance the ability of DCs to present antigens to T cells and regulate T lymphocyte activation.	[29]
	BMMC-derived exosomes are able to lower IgE levels and block mast cell activation.	[97]
	MC-derived EVs convey a protective message under oxidative stress, decreasing mortality.	[98]
	miR-21 released from MC-derived exosomes enhances oxidative stress and triggers inflammatory reactions in asthmatic mice.	[94]
	Exosomes activated by IgE from MCs exacerbate atherosclerosis by inducing endothelial dysfunction through the circular RNA CDR1as, linking asthma with atherosclerosis.	[99]
**Dendritic cells**	DC-derived exosomes can activate allergen-specific Th2 cells.	[29]
	DC exosomes specifically activate OVA-targeted CD8+ T cells and promote OVA-specific IgG antibody production.	[104]
	DC-derived exosomes carry enzymes necessary for producing leukotrienes.	[105,106]
	DC-derived exosomes, when packed with chemotactic eicosanoids, promote inflammation and granulocyte migration in vitro.	[106]
	Various subsets of pulmonary DCs have been identified, each contributing differently to asthma pathogenesis.	[107,108]
**Neutrophils**	Neutrophil-derived exosomes play a role in regulating changes in airway smooth muscle structure.	[113,114]
	OVA-induced airway epithelium-derived exosomes increase AHR and trigger the accumulation/activation of macrophages, neutrophils, and eosinophils.	[115]
	Neutrophil-derived EVs disrupt epithelial cell connections.	[116]
	Exosomes released by neutrophils contribute to airway structural changes, promoting the migration and proliferation of ASMCs in response to LPS.	[117]
	Neutrophil-derived exosomes containing elastase contribute significantly to airway inflammation.	[118]
**Macrophages**	Macrophage-derived exosomes have a significant impact on T1 immune reactions.	[126,127]
	In SSRA, M1 macrophages release high levels of inflammatory molecules, contributing to neutrophil-rich infiltration, AHR, and airway structural changes.	[128]
	Exosomes secreted from human macrophages have a proinflammatory role in asthma and contain enzymes that favor LTC4 production.	[106]
	Exosomes secreted by M2 macrophages reduce lung inflammation and asthma progression through the action of miR-370.	[129]
**Mesenchymal stem cells**	MSC-EVs exhibit therapeutic effects similar to MSCs but with reduced risks of immune rejection, tumorigenicity, and pulmonary embolism.	[131]
	EVsderived from mesenchymal stem cells have a similar therapeutic effect to their parental cells.	[132]
	EVsderived from mesenchymal stem cells impact immune cells and inhibit airway remodeling.	[13]
	ASCs and other MSCs can reduce allergic airway inflammation in bronchial asthma mouse models.	[133]
	ASC-derived EVs immunomodulatory effects are thought to involve the suppression of Th2 cytokine production in airway allergic inflammation.	[134]
	AD-MSC-derived exosomes showed beneficial effects on ovalbumin-induced allergic asthma.	[135]
	MSC-derived extracellular vesicles have been proposed as a promising alternative to MSCs for treatments such as asthma.	[136]
	BMMC-derived exosomes highly expressed a specific miRNA, miR-223-3p, known to be associated with high inflammation and the exacerbation of asthma.	[137]
	MSC-derived exosomal miR-1470 induces the expression of P27KIP1 in asthmatic patients, promoting the differentiation of CD4+CD25+FOXP3+ Tregs.	[138]
	BMMC-derived exosomes contain miR-188, which has a negative effect on airway remodeling and lung injury.	[139]
	hUCMSC-derived EVs have a therapeutic effect in SSRA, with an action on the NF-kB and PI3K/AKT signaling pathways.	[140]
	Migrasomes secreted from hUCMSCs play a role in the protective effect of hUCMSCs in asthma.	[141]
	Hypo-EVs are able to reduce airway inflammation and remodeling in asthmatic mice.	[142]
	Hypo-EVs have a therapeutic effect on epithelial barriers both in vivo and in vitro.	[143]
	The therapeutic mechanisms of MSC-EVs can be categorized into several key pathways in the context of asthma treatment.	[144]

## 4. Methods for EVs Analysis: Available Tools, Potential, and Challenges

**Figure 3:**
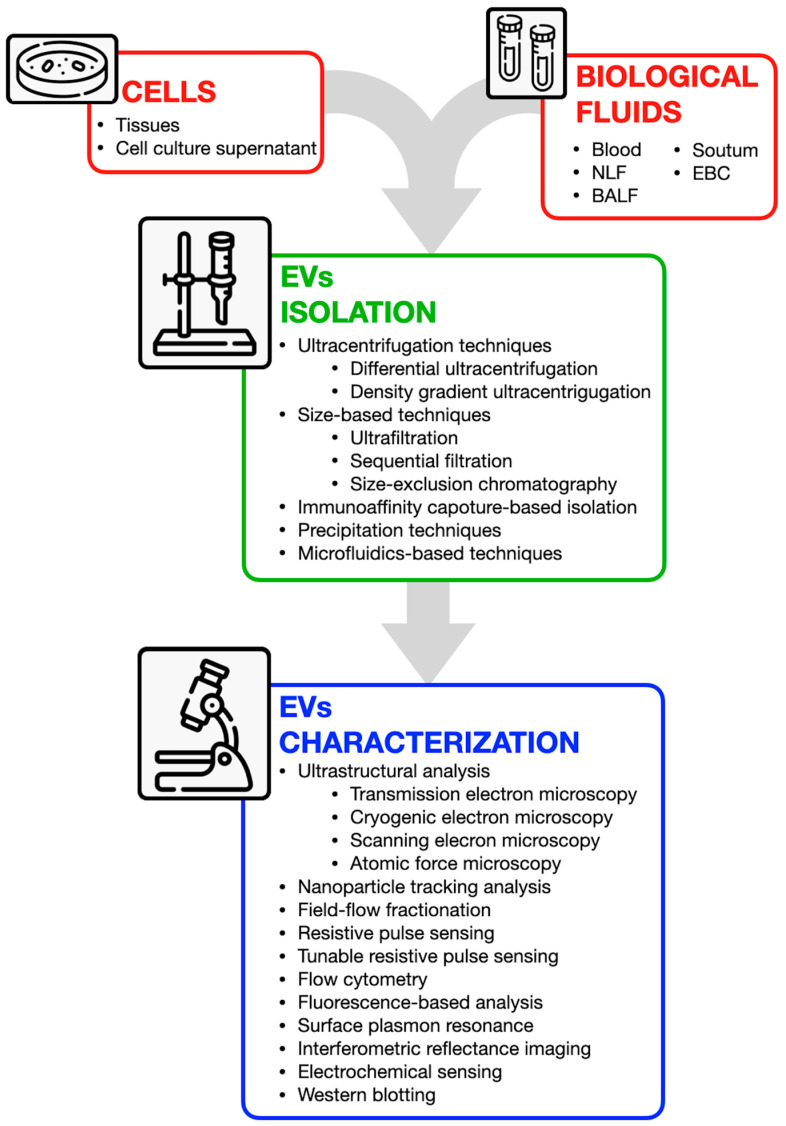
Overview of available techniques for extracellular vesicle isolation and characterization. EVs can be obtained from various sources, including cells (from tissues or culture supernatants) and biological fluids (e.g., blood, nasal lavage fluid, bronchoalveolar lavage fluid, sputum, and exhaled breath condensate). Isolation techniques include ultracentrifugation, size-based separation, immunoaffinity capture, precipitation, and microfluidics-based approaches. Characterization methods encompass ultrastructural analysis (e.g., electron microscopy), nanoparticle tracking, flow cytometry, fluorescence-based assays, and antibody-based techniques such as Western blotting.

A crucial step in understanding EVs’ biological roles is their efficient extraction and enrichment. As research on EVs progresses, their potential applications are becoming increasingly evident. Exosomes have the potential to serve as powerful biomarkers and therapeutic agents for early disease diagnosis, monitoring treatment responses, and predicting prognosis. For large-scale clinical application, key requirements include rapid and straightforward isolation methods, high yield, purity, reliable characterization, safety, cost-effectiveness, and therapeutic efficacy. However, identifying exosomes remains challenging due to their variability in size, composition, function, and origin. Different isolation techniques are utilized based on the specific application and objective [145]. The most commonly employed methods include immunoaffinity capture, polymer precipitation, size-based isolation techniques, and ultracentrifugation, as illustrated in Figure 3. Depending on factors such as sample type, environmental conditions, and vesicle abundance, EVs can be extracted from various sources, including bodily fluids, solid tissues, and cell culture media, using different isolation techniques. The optimal method for isolation should be selected based mainly on the specific source (bodily fluid, tissue, cell culture supernatant) and on the further application, such as cargo analysis, basic research, clinical applications, etc. Key factors to consider include the sample type, equipment availability, yield, purity, and efficiency in terms of time and effort. An ideal isolation technique should be simple, rapid, cost-effective, and capable of producing high-purity and high-yield exosomes without altering their natural structure or requiring expensive equipment. While each method has its strengths and limitations, improvements through protocol modifications or combining techniques could enhance EV research for both fundamental and therapeutic applications. A complete description of all the available methods is beyond the scope of our review and can be accessed elsewhere [146]. Nevertheless, for the isolation of cell culture model-derived EVs, for example, including both eukaryotic and prokaryotic cells, various methods have been developed, each with their own advantages and limitations. Since many extracellular particles share similar properties with exosomes, most isolation techniques focus on separation based on size and density. Initially, cells and debris are removed from the culture medium through sequential centrifugation at low speed. The resulting supernatant, which contains exosomes, is then transferred to a new tube for further processing using the chosen isolation method or stored for future use. Although the physiological environment in vivo differs from that of cell culture, using cell culture media as a source of EVs allows for more controlled conditions during EV production. However, due to the challenge of eliminating contaminating serum exosomes, other EVs, proteins, and lipoproteins, it is advisable to culture cells in a chemically defined medium when highly pure exosomes are required for omics analysis or functional studies. To ensure accuracy and prevent contamination, strict procedural guidelines should be followed during vesicle isolation [147,148].

After isolation and enrichment, EVs are usually characterized (in terms of size and concentration) by microscopy and optical/physical methods such as transmission electron microscopy (TEM) and all its variants as well as nanoparticle tracking analysis (NTA). Western immunoblotting is also employed to detect EV-related biomarkers such as tetraspanins. EV cargo analysis can then be achieved by several approaches including NGS, MS, and antibody-based methods. Surface antigens analysis by flow cytometry also represents an interesting way of assessing EVs’ characteristics, highlighting the peculiar markers of originating cells and the precise molecules needed for targeting the EVs to the recipient cells. For updated information on best practices in EVs research, one can refer to the latest guidelines provided by the International Society for Extracellular Vesicles (ISEV) [149]. As the research in this field rapidly advances, new methodologies are expected to facilitate their translation into therapeutic applications. However, current isolation and analysis methods often require costly equipment and specialized expertise. Future advancements in exosome/EVs technology will focus on improving preservation, detection sensitivity, and resolution to enable more efficient separation and application. Next-generation techniques should be faster, more sensitive, cost-effective, and reliable. Despite challenges in isolation, purification, characterization, large-scale production, the standardization of operation methodologies, potential immune response, and drug loading, their unique properties make them promising for early disease diagnosis and treatment.

## 5. Discussion

Bronchial asthma is a complex disease which has been extensively studied in recent years. Despite increasing knowledge about immunological mechanisms, disease phenotyping in everyday clinical practice is still limited by the relatively poor accuracy of easily available biomarkers. They include blood eosinophils, IgE, and exhaled nitric oxide in the case of T2-high asthma or the absence of those biomarkers in T2-low phenotypes. Such simplification does not reflect the complex background underlying clinical manifestations. Therefore, a targeted therapy selection process is also hampered.

On the contrary, EVs can be considered a much more accurate source of information about the immunological activation. In fact, the molecular structures expressed on their surface can be related to the cells where EVs come from and where they are moving to. In addition, the reach repertoire EVs contain provides some details about the kind of “message” they are carrying out within the context of the ongoing inflammation.

Among the molecules carried by EVs, non-coding-RNA are the most abundant. Exosome cargo is also characterized by a variety of non-coding RNAs, including miRNAs and long non-coding RNA [36,150]. Furthermore, miRNAs were earlier described as associated with asthma pathogenesis and response to treatment [30]. Elevated levels of MiR-21 were detected in serum samples of patients affected by eosinophilic asthma as compared to healthy individuals. MiR-21 stimulates Th2 responses by inhibiting IL-12 gene expression and decreasing the release of IFN-γ, which leads to elevated Th2 cytokines. Moreover, it can induce the differentiation of T cells through action on Gata3 and IL-4 [151]. In recent years, it was shown that miR-223 had elevated expression levels in asthma [134], and at exosomal levels, miR-223 was upregulated in subjects with asthma as compared to healthy controls [152]. Moreover, a recent study utilizing a logistic regression model revealed that the collective expression of miR-21-5p, miR-126-3p, miR-146a-5p, and miR-215-5p extracted from serum exosomes could distinguish between levels of asthma severity. In particular, miR-21-5p and miR-126-3p showed an overexpression in bronchial epithelial cells that correlated with the induction of IL-13 production [153]. MiR-146a-5p, also expressed in the bronchial epithelium, is induced by different cytokines such as TNF-α, IL-4, and IL-17A, and it is correlated with the Nf-kB pathway [154]. MiR-21-5-p and miR-126-3pm levels were found to be increased in type 2 high atopic asthma, and a significant decrease in miR-21-5p, miR-126-3p, and miR-146a-5p corresponded with the IL-6 high endotype, obesity, or neutrophilic asthma [153].

EVs’ role in asthma and other respiratory diseases has been widely assessed by using biological fluids as a source [15]. Serum, plasma, nasal lavage fluid, sputum, and bronchoalveolar fluids have been used as they are easily accessible and allow a broader range of information to be gained on the function of EVs. According to the evidence mentioned above, EV analysis has the potential to pave the way to a new perspective on asthma endotyping by uncovering the communication among immune and epithelial cells. In particular, EVs could be helpful in the understanding of the inflammatory mechanisms that could drive inflammation in different asthma endotypes. Taken together, the available evidence supports the relevance of EVs as valuable markers of asthma molecular profile and severity. On the other hand, they remain quite limited to the research setting as they require specific tools and expertise to be measured and interpreted. Ensuring the safety and purity of exosome samples is essential, especially in research and medical applications. When used in medical treatments, exosomes must undergo rigorous safety testing and comply with all regulatory standards before being administered to patients [155]. As a further, future perspective, EVs could be considered potential targets for new extremely selective therapeutic approaches.

## 6. Conclusions

Traditional Th1/Th2-based phenotyping, especially when relying on routinely available biomarkers, lacks accuracy in capturing the complexity of the immunological background underlying asthma pathobiology. In a clinical context, it might hamper asthma patients’ correct classification and consequently the treatment selection process. EVs can be considered as dynamic biomarkers providing the unique opportunity to “track” the cell-to-cell cross-talk which is ongoing at the time of observation [156]. Under a pathobiological perspective, EV assessment might pave the way to a better understanding of asthma inflammation and to an innovative way of endo-phenotyping besides traditional Th1/Th2 labels and related biomarkers [138]. At the moment, a major limitation is represented by the applicability of EVs assessment in everyday clinical practice. This is basically related to the lab procedures required for EVs investigation and to the lack of standardized reference values validated on large population samples.

Additionally, due to the complexity of their biogenesis and heterogeneity, further research is needed to fully understand their biological functions and therapeutic potential. To maximize their clinical benefits, future advancements should focus on enhancing targeted delivery, developing scalable and reproducible isolation methods, and refining exosome engineering techniques.

However, although still limited to translational research, EVs exploration seems a promising field for advancing the knowledge on the disease mechanisms and potentially highlighting therapeutic targets.

## Data Availability

No new data were created or analyzed in this study. Data sharing is not applicable to this article.

## Abbreviations

The following abbreviations are used in this manuscript:

AD-MSCs	Adipose tissue-derived mesenchymal stromal cell
AERD	Aspirin-exacerbated respiratory disease
AHR	Airway hyperresponsiveness
APC	Antigen-presenting cell
ASCs	Adipose-derived stem cells
ASM	Airway smooth muscle
BALF	Bronchoalveolar lavage fluid
BECs	Bronchial epithelial cells
BMMCs	Bone marrow-derived mesenchymal stem cells
BSMCs	Bronchial smooth muscle cells
circ	Circular
cysLT	Cysteinyl leukotrienes
DCs	Dendritic cells
DRMs	Detergent-resistant membrane microdomains
ECP	Eosinophil cationic protein
EDN	Eosinophil-derived neurotoxin
EPX	Eosinophil peroxidase
ETM	Epithelial-to-mesenchymal transition
EVs	Extracellular vesicles
FeNO	Nitric oxide
FGM1	Fibroblast growth factor 1
GM-CSF	Granulocyte–macrophage colony-stimulating factor
hUCMSCs	Human umbilical cord mesenchymal stem cells
Hypo-EVs	Hypoxic hUCMSC-EVs
IFN-γ	Interferon-gamma
IgE	Immunoglobulin E
ISEV	International Society for Extracellular Vesicles
LAMP1	Liposomal-associated membrane protein 1
LFA1	Lymphocyte function-associated antigen-1
lnc	Long noncoding
LPS	Lipopolysaccharide
MBP	Major basic protein
MCs	Mast cells
MHC	Major histocompatibility complex
miRNAs	MicroRNAs
MSC-EVs	MSC-derived extracellular vesicles
MSCs	Mesenchymal stem cells
NHBE	Normal human bronchial epithelial cells
NO	Nitric oxide
NTA	Nanoparticle tracking analysis
PDCs	Plasmacytoid
PS	Phosphatidylserine
ROS	Reactive oxygen species
SSRA	Severe steroid-resistant asthma
TCR	T cell receptor
TEM	Transmission electron microscopy
TF	Tissue factor
Tregs	Regulatory T cells
UCMSC-EVs	Umbilical cord mesenchymal stem cell-derived EVs
